# X-Ray Investigation of Strain in Cold-Worked Silver Iodide*

**DOI:** 10.6028/jres.068A.034

**Published:** 1964-08-01

**Authors:** G. Burley

## Abstract

The experimentally observed broadening of certain powder diffraction profiles of cold-worked hexagonal silver iodide has been graphically resolved into the effects due to small domain size and to lattice strain. The contribution from the first was found to be negligible, and the total intrinsic broadening is thus due to strain. A maximum, or saturation value, of strain is rapidly attained and remains invariant with further grinding. The strain is essentially isotropic, with an average value of 4.4×10^−3^. The calculated stored elastic energy is 154 cal/mole and, by comparison with the calculated difference in lattice energies of 149 cal/mole, can be interpreted as an energy barrier for the phase transition from the cubic to hexagonal structure.

## 1. Introduction

Cold-working or plastic deformation of solids can produce appreciable changes in the intensity distribution of diffracted x rays [1–3]. One of the most prominent of these effects is a change in line shape, specifically line broadening. This broadening may be due to one or more of three factors: very small coherently diffracting domains, randomly directed and slowly varying internal lattice strains, or stacking faults.

The various causes of line broadening can be separated under favorable circumstances by either a Fourier analysis of line shapes or an analytical study of line breadths and peak shifts. In the absence of higher order reflections the second technique, in addition to its relative simplicity, offers certain other advantages. These include especially the ability to separate easily the effects of small particle size and lattice strain. It has been shown [4] that the integral line breadth (in radians) caused by strain is

$$\beta_{s}=2\xi \;\tan\;\theta .$$ (1)

where ξ represents the strain distribution and *θ* the Bragg angle, and that the line breadth resulting from small particle size only is given by

$$\beta_{p}=\lambda /L\;\cos\;\theta$$ (2)

where λ is the x-ray wavelength and *L* is a mean linear dimension of the particles designated the apparent particle size. In the presence of both strain and small particle size line broadening the composite broadening function is approximately

β=2ξtanθ+λ/Lcosθ. (3)

This composite broadening depends to some extent on the broadening functions of the separate effects. Only if both broadening functions are Cauchy curves are the breadths strictly additive [5].

For computational purposes the above formula can be put in the form

$$\frac{\beta \;\cos\;\theta }{\lambda }=\frac{2\xi \;\sin\;\theta }{\lambda }+\frac{1}{L}.$$ (4)

A plot of β^*^ (*= β* cos *θ*/λ) showing the simultaneous effects of small particle size and strain broadening against d* (= 2 sin *θ*/λ) will then be linear with a slope of ξ and intercept 1*/L.* If both functions are not Cauchy curves, this will result in deviations from linearity.

Silver iodide can exist at room temperature in both the cubic and hexagonal close-packed structures. In this respect it is very similar to cobalt, where the isotropy of the lattice strain for the hexagonal modification has recently been reported [6]. The ease of plastic deformation of silver iodide by cold-working allows the study of this effect on a binary inorganic compound by similar techniques. The aim of this paper will be to report the results obtained from an analysis of the broadening of certain diffraction lines of this compound due to grinding, with emphasis on a study of the distribution of strains in the case of a deformed hexagonal structure.

## 2. Experimental Procedure

Polycrystalline silver iodide was prepared by slow addition of a solution of silver nitrate to one of sodium iodide. The powder was filtered, air-dried, and held at 125 °C for four weeks. This resulted in a mixture containing approximately 70 percent of the hexagonal phase. A portion of this sample was then ground vigorously until approximately 95 percent was converted to the cubic phase. Powder x-ray diffraction patterns were taken for each specimen on a high-angle x-ray diffractometer with manganese filtered iron, nickel filtered copper, and zirconium filtered molybdenum radiations.

Sharp diffraction profiles were obtained for the well-annealed specimens in each case, but the profiles for the cold-worked specimens showed definite broadening. This broadening was roughly proportional to the amount of deformation until maximum conversion occurred, and then remained essentially constant with further grinding.

In order to eliminate the effects of stacking faults only those reflections common to both the face-centered cubic and the hexagonal low-temperature phases of silver iodide were used in this analysis. The almost complete absence of higher order reflections precluded an accurate study by the method of Fourier analysis of line profiles and the experimental techniques associated with the direct study of the observed peak shapes were therefore employed here.

The half-height peak widths were taken as the experimental breadths of the diffraction lines. This angular width at half-maximum intensity is here designated *b* for the annealed specimens, and *B* for the cold-worked specimens. The pure intrinsic x-ray diffraction broadening derived from these is designated *β.* Experimental line breadths were corrected graphically both for the effects of *Kα*-doublet separation and instrumental broadening [7]. The data leading to the determination of the pure intrinsic broadening *β* are given in table 1.

## 3. Results

**Figure 1:**
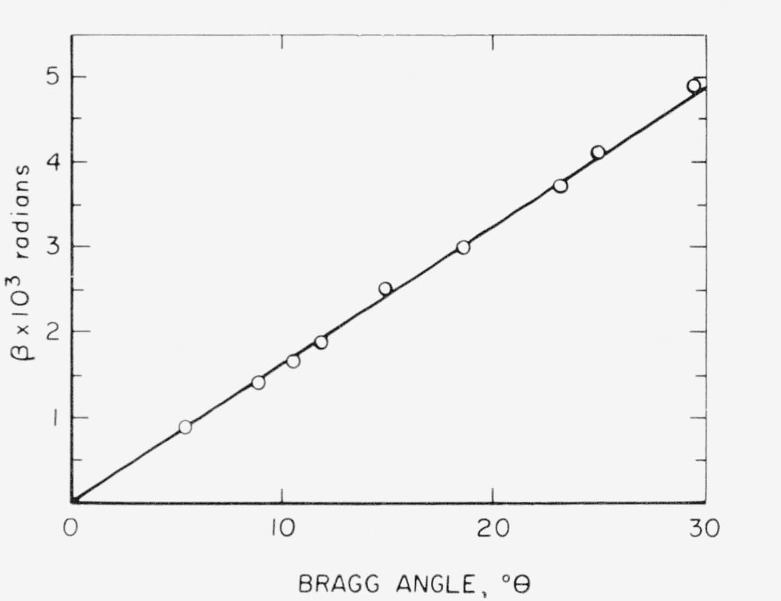
The intrinsic broadening function for cold-worked silver iodide as a function of the Bragg diffraction angle.

**Figure 2:**
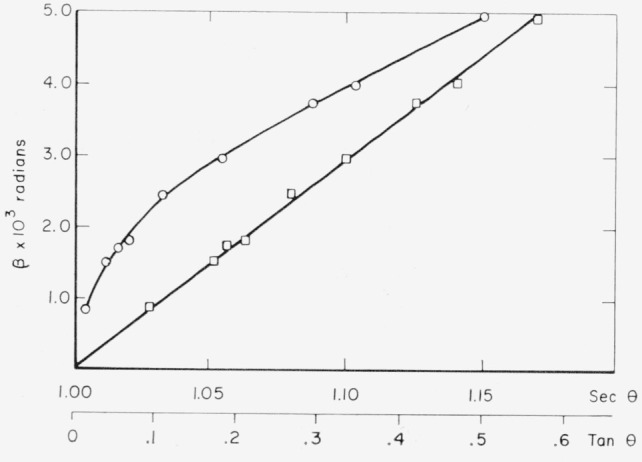
The variation of the intrinsic broadening function for silver iodide with tan θ (squares) and sec θ (circles).

Values of the pure diffraction broadening for silver iodide are shown in figure 1 in terms of *β* expressed in radians versus the Bragg angle in degrees *θ*. The variation of *β* with respect to tan *θ* and sec *θ* is shown in figure 2. The linearity of the tan *θ* plot, opposed to the pronounced curvature at low angles of the sec *θ* plot, suggests agreement with the criteria of pure strain broadening for silver iodide.

**Figure 3:**
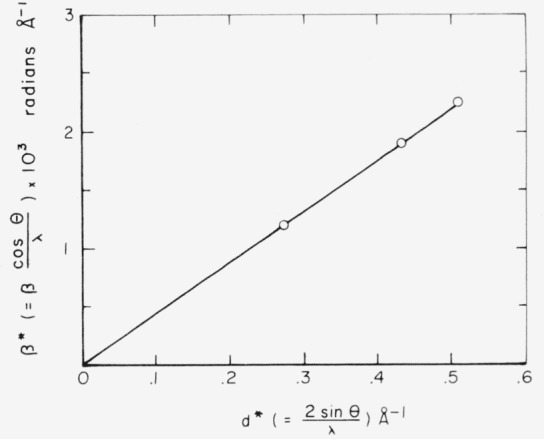
The relation of β^*^ and d^*^ for the (002), (110), and (112) reflection of cold-worked silver iodide.

Effects of strain and small particle size on the total broadening function were resolved by a plot of *β* cos *θ*/λ against 2 sin *θ*/λ. This is shown in figure 3. Averaged values of the first term for all three wavelengths were used, the maximum deviation being 0.02 radians Å^−1^. All experimental points fall on a straight line with zero intercept, indicating that the total broadening is due to lattice strain alone. Numerical values of the strain derived from the slope, as well as that calculated from each experimental value by means of eq 1 are shown in table 2. The strain is isotropic, within the limits of error of the measurements.

The choice of only those reflections for which the broadening is independent of the stacking fault contribution effectively eliminates this contribution. The entire observed line broadening in cold-worked silver iodide for these reflections only is thus due to lattice strain. No attempt was made to estimate the number of stacking faults, nor the correlation of these with the structural transformation.

## 4. Stored Elastic Energy

Both elastic and plastic deformation produce changes in the internal energy of a solid. Plastic deformation by cold work may require the expenditure of much larger amounts of energy than elastic deformation, but only a small fraction of this energy is retained in the lattice. This increase in internal energy caused by cold work is termed the stored energy. Cold work also changes the free energy of the deformed material. This change in free energy is the true measure of the thermodynamic instability induced by deformation, but it is the change in internal energy which is actually measured in investigations of stored energy. For most purposes it is convenient to equate the free and internal energies of cold work [8].

In the absence of fragmentation and stacking fault effects, the strain energy derived from x-ray line broadening results can be identified with the total stored energy. However, for a, variety of reasons, the values obtained by the x-ray method have often been lower. An exact correlation is therefore not to be expected.

On the basis of an isotropic stress distribution, the stored energy *V* has been related by Faulkner [9] to the average strain *e* and Young’s modulus *E*, by

$$\begin{array}{c} V=\frac{15E}{4\left(1+v\right)}\epsilon^{2}\\ =2.78E\epsilon^{2} \end{array}$$

evaluated for the Poisson’s ratio *v* = 0.35 of silver iodide.

A value for Young’s modulus for silver iodide of 9.086×10^10^ dyne cm^−2^ at room temperature was determined by J. B. Wachtman [10] of this laboratory by extrapolation to zero porosity of the results on two cold-pressed specimens. This, together with the average strain determined in this work, gives a calculated value of the stored elastic energy for cold-worked silver iodide of 0.66 cal g^−1^ or 154 cal mole^−1^, with an estimated uncertainty of ±10 cal mole^−1^.

## 5. Conclusions

It has been shown that the elastic strain distribution for hexagonal silver iodide is essentially isotropic. This is in agreement with the value expected for the ideal close-packed structure with an axial ratio of 1.667. The transition of the hexagonal to the close-packed cubic phase induced by grinding is always accompanied by strain. Annealing between 90° and 120 °C then leads to strain relief with negligible reversal to the hexagonal phase.

The stored energy of cold work is of approximately the same magnitude found for some metals after grinding or rolling [8]. It rapidly approaches the maximum value reported and then remains approximately constant for varying grinding times. This indicates that saturation strain is rapidly attained, which may perhaps be interpreted in terms of an energy barrier for the phase transition. The corresponding difference in lattice energies for the two phases, calculated from coulomb potentials derived from the respective Madelung constants, is 149 cal mole^−1^.

## Figures and Tables

**Table 1:** Experimental data and intrinsic line breadth for cold-worked silver iodide

Radiation	*hkl*	*θ*	*d* (°2*θ*)	*B*_o_ (°2*θ*)	*B* (°2*θ*)	*b*_o_ (°2*θ*)	*b* (°2*θ*)	*b/B*	*β/B*	*β*	
										(°2*θ*)	(radians)
											
Mo	002	5.42	0.066	0.24	0.22	0.20	0.17	0.77	0.25	0.052	0.90×10^−3^
	110	8.93	.112	.24	.16	.18	.09	.56	.51	.082	1.45
	112	10.43	.130	.24	.14	.17	.06	.43	.68	.096	1.70
Cu	002	11.85	.064	.23	.21	.16	.12	.58	.50	.105	1.85
	110	19.60	.096	.28	.23	.15	.08	.35	.75	.172	3.00
	112	23.15	.124	.32	.25	.16	.06	.24	.86	.214	3.72
Fe	002	14.95	.066	.25	.24	.15	.12	.49	.61	.146	2.50
	110	24.94	.112	.33	.27	.15	.06	.22	.88	.237	4.12
	112	29.61	.140	.35	.28	.14	0	0	1.00	.280	4.90

*θ*=Bragg angle

*d*=separation of K*α*_1_−*α*_2_ doublet

*B*_o_=observed half-width line breadth for cold-worked specimen

*B=B*_o_ corrected for effects of K*α*-doublet separation

*b*_o_=observed half-width line breadth for annealed specimen

*b=b*_o_ corrected for effects of K*α*-doublet separation

*β*=pure diffraction breadth corrected for both the K*α*-doublet separation and instrumental broadening.

**Table 2:** Strain for silver iodide

*hkl*	Radiation	*ϵ* (×10^3^)	
			
002	Mo	4.46	
	Cu	4.41	
	Fe	4.62	av=4.50×10^−3^
110	Mo	4.15	
	Cu	4.46	
	Fe	4.44	av=4.39×10^−3^
112	Mo	4.61	
	Cu	4.35	
	Fe	4.43	av=4.48×10^−3^ overall av=4.46×10^−3^ calc from slope of fig. 3, *ϵ* = 4.44×10^−3^
